# Mortality, Length of Stay and Cost of Hospitalization among Patients with Adult-Onset Still’s Disease: Results from the National Inpatient Sample 2016–2019

**DOI:** 10.3390/diseases12070166

**Published:** 2024-07-22

**Authors:** Sushmita Mittal, Benjamin Schroeder, Musaab Alfaki

**Affiliations:** Department of Medicine, University of Missouri, Columbia, MO 65212, USA

**Keywords:** adult-onset still’s disease, in-hospital mortality, hospital charges, length of stay

## Abstract

We use this study to analyze the trends in in-hospital length of stay, total hospital charges, and mortality among adult patients with a primary diagnosis of adult-onset still’s disease (AOSD). We used the 2016–2019 National Inpatient Sample (NIS) database to conduct a retrospective study on adult AOSD patients (≥18 years old). We analyzed data on baseline patient and hospital characteristics and determined trends in in-hospital mortality, length of stay (LOS), and total hospital charges (TOTCHG). Univariate and multivariate linear and logistic regression analyses were performed to identify factors that independently affected these outcomes. Among the 1615 AOSD hospitalizations, the mean LOS was 7.34 days and the mean TOTCHG was 68,415.31 USD. Macrophage activating syndrome (MAS), disseminated intravascular coagulation (DIC), and a large hospital size were shown to statistically increase the LOS and TOTCHG, while a Native American background was shown to statistically decrease both. The mean in-hospital mortality was 0.929%, with age being the only independent predictor. Our findings reveal an increase in the economic burden of AOSD hospitalizations despite declining admissions and mortality rates. Complications, like MAS and DIC, were found to significantly contribute to this burden despite treatment advancements. Our study indicates the importance of investigating new strategies to prevent these complications.

## 1. Introduction

Adult-onset Still’s disease (AOSD) is a rare multi-systemic inflammatory disorder that occurs due to innate immune cell activation. It is a severe version of juvenile idiopathic arthritis, which occurs in children. Currently, AOSD is diagnosed using the Yamaguchi criteria. This scoring system requires at least five features to be present to diagnose patients with AOSD, two of them being major characteristics. Major criteria include fever, arthralgias, an evanescent salmon-colored rash, and leukocytosis. Minor criteria include sore throat, lymphadenopathy, increased serum aminotransferase or lactate dehydrogenase, and negative immunoglobulin M rheumatoid factor and antinuclear antibodies. The diagnosis of AOSD also requires the exclusion of other diseases that present with overlapping clinical features, including malignancy, infections, and other autoimmune disorders [1]. Currently, AOSD has an incidence of 0.16–0.4 per 100,000 adults in the United States (US). It has a bimodal age distribution, with peaks at ages 15–25 years and 36–45 years [2].

While there are a few studies that have analyzed in-hospital trends among AOSD patients, most of these studies are derived from small sample sizes that limit the generalizability of the results. To the best of our knowledge, the largest nationwide population-based study on AOSD patients in the Unites States was performed using in-hospital data from NIS 2009–2013. Mehta’s study revealed that the average length of stay was 6.9 days, the average total hospital charges were 30,857 USD, and the number of in-hospital deaths was 154 over the 4-year period [3]. Since then, there have been several advancements in AOSD treatment and management that can impact in-hospital mortality, total charges, and length of stay among patients with AOSD. In this study, we use the 2016–2019 National Inpatient Sample (NIS) database to perform a comprehensive analysis of these inpatient trends.

## 2. Materials and Methods

### 2.1. Data Source

We conducted a retrospective cohort study using the 2016–2019 National Inpatient Sample (NIS) database, the largest publicly available database of hospitalizations in the US. This database was developed for the Healthcare Cost and Utilization Project (HCUP). It contains de-identified data, including demographics, discharge diagnoses, procedures, hospital charges, comorbid conditions, and outcomes, from a sample of more than 7 million (unweighted) hospital stays each year. The diagnoses and procedures used in this study were identified using the International Classification of Diseases, Tenth Edition, Clinical Modification/Procedure Coding System (ICD-10 CM/PCS). All the patient information in our study was de-identified and, therefore, did not require institutional review board approval. Informed consent and ethics approval were also not required for our submission as we have de-identified all patient data for this case.

### 2.2. Analytic Sample

The study population included adults aged 18 and older with a primary diagnosis of AOSD, identified using the ICD-10 code M06.1. Patients with missing demographic data and an age < 18 years old were excluded from this study. AOSD typically does not overlap with other rheumatic diseases, so we also excluded patients with a concurrent diagnosis of Systemic Lupus Erythematosus, Systemic Sclerosis, Polymyalgia Rheumatica, Psoriatic Arthritis, Rheumatoid Arthritis, Ankylosing Spondylitis, and Dermato-polymyositis (ICD-10 codes provided in Table 1). Patients with adult transition cases of systemic juvenile idiopathic arthritis (SJIA) were also included in this study if they were assigned a primary diagnosis of AOSD with ICD-10 code of M06.1 during their hospital stay. All acronyms used in this paper have been outlined in Table 2.

Patients who had a secondary diagnosis of AOSD were not included in the analysis as they can be hospitalized for reasons that are not related to their AOSD diagnosis which can skew the results. It is not possible to identify patients with a secondary diagnosis with AOSD who were admitted due to a complication of AOSD, so all patients with a secondary diagnosis of AOSD were eliminated from our study.

### 2.3. Variables

The variables were divided into patient level, illness severity, and hospital-level. Patient level variables included age, sex, race, co-morbidities, and median income quartile based on the patient’s zip code.

Illness severity variables included in-hospital mortality, in-hospital length of stay (LOS), in-hospital total charge (TOTCHG), macrophage activation syndrome (MAS), thrombotic thrombocytopenic purpura (TTP), and disseminated intravascular coagulation (DIC). The total hospital charge, analyzed in this study, reflects the hospital bill for the entire hospital stay and does not include physician fees and non-covered charges.

Hospital level variables included bed size, location and teaching status, and region (Northeast, South, Midwest, and West). Bed size was further divided into small, medium, and large and are specific to the hospital’s urban–rural designation and teaching status. Our study used the hospital bed size categories that were defined after 1998. Further details on the bed size can be found on the following website: https://hcup-us.ahrq.gov/db/vars/hosp_bedsize/nisnote.jsp, accessed on 23 May 2023. The hospital location was divided into urban and rural locations which was based on the Core Based Statistical Area Codes (CBSA). Hospitals that were in areas with a CBSA type of “Division” or “Metro” were considered urban while those with a CBSA type of “Rural” or Micropolitan” were classified as rural. In our study, we combined the location and teaching status in one variable which was divided into rural, urban non-teaching, and urban teaching. Further information regarding this can be found on: https://hcup-us.ahrq.gov/db/vars/hosp_locteach/nisnote.jsp, accessed on 23 May 2023.

### 2.4. Study Outcomes

The primary outcome of interest was analysis of the trends of in-hospital mortality rate, length of stay, and total charge in patients with AOSD. We also highlighted the independent predictors of these in-hospital outcomes after adjusting for other variables.

### 2.5. Statistical Analysis

STATA software version 17 (Stata Corporation, College Station, TX, USA) was used to conduct the data analysis. To ensure national estimates, we performed our data analysis using weighted samples that adhered to HCUP requirements for using the NIS database. Baseline hospital and patient characteristics for AOSD hospitalizations were derived using Student’s t-test for continuous variables and Pearson’s Chi-square test for categorical variables. Independent predictors for TOTCHG and LOS were determined using univariate and multivariate linear regression analyses. Univariate and multivariate logistic regression analyses were used to assess independent predictors of mortality among AOSD patients. The variables that were adjusted for in the analysis included age, gender, race/ethnicity, median yearly income based on the patient’s zip code, the patient’s co-morbidities (using Elixhauser Comorbidity Index), hospital bed size, hospital location and teaching status, and hospital region. A *p*-value of <0.05 was considered significant for our study.

## 3. Results

### 3.1. Patient and Hospital Characteristics

A total of 1615 patients were hospitalized between January 2016 and December 2019 with a primary diagnosis of AOSD. The patients were predominantly female (60.68%) and White (50%), with a mean age of 42.54 years. Most patients with AOSD were treated in large hospitals (66.56%) that were classified as urban teaching (86.07%). Further baseline characteristics of AOSD patients can be seen in Table 3.

### 3.2. Analysis of Trends and Independent Predictors of In-Hospital LOS

AOSD patients were found to have a mean LOS of 7.34 days. The odds of a longer hospital stay increased with MAS (adjusted odds ratio (aOR): 8.64, *p* = 0.017), DIC (aOR: 8.07, *p* = 0.002), and large hospital size (aOR: 2.49, *p* = 0.007). In contrast, Native American race was associated with a lower adjusted length of stay (aOR: −2.79, *p* = 0.000) compared to White patients. Age, female sex, TTP, and other races (Black, Hispanic, Asian or Pacific Islander, and others) did not have any individual impact on length of stay on both univariate and multivariate analyses. Further details can be found in Table 4.

### 3.3. Analysis of Trends and Independent Predictors of In-Hospital TOTCHG

From 2016–2019, AOSD patients were found to have a mean in-hospital TOTCHG of 68,415.31 USD. Similar to the LOS trends, our results revealed that MAS, DIC, Native American race, and large hospital bed size were the only independent factors that affected in-hospital TOTCHG. The odds of higher TOTCHG increased with MAS (aOR: 106,711.80 USD, *p* = 0.033), DIC (aOR: 70,001.37 USD, *p* = 0.000), and large hospital size (aOR: 29,572.07 USD, *p* = 0.011). In contrast, the adjusted TOTCHG decreased with Native American race (aOR: 47,372.49 USD, *p* = 0.016) when compared to White patients. Further details can be found in Table 5.

### 3.4. Analysis of Trends and Independent Predictors of In-Hospital Mortality

From 2016–2019, there were only 15 patients with a primary AOSD diagnosis who experienced in-hospital mortality. Further analysis revealed that all 15 deaths occurred in the 18–35 years old age group and none in the >35 years old age group. After adjusting for other variables, our results revealed that, for every 1-year increase in age, there is an 8% decrease in the odds of mortality. In contrast, MAS, DIC, TTP, female sex, and race were not independent predictors of in-hospital mortality (aOR: 1). Further details of these findings are described in Table 6.

## 4. Discussion

Several studies have investigated LOS and TOTCHG among hospitalized AOSD patients; however, to the best of our knowledge, none have analyzed their independent predictors. Our data found that the average LOS for AOSD patients was 7.34 days, which was comparable to Mehta’s 2009–2013 NIS study, which revealed an average LOS of 6.9 days. In contrast, our data revealed a substantial increase in the mean total charge (68,415.31 USD) compared to Mehta’s study (30,857 USD) [3]. MAS and DIC are well-known complications of AOSD and were found to be independent predictors of increased LOS and TOTCHG among AOSD patients. This could be due to the decrease in the AOSD mortality rate seen in our study (0.92%), which can come at the expense of increased complications associated with the progression of AOSD, including DIC and MAS. Another explanation for this increase in TOTCHG and LOS with MAS could be due to the increased diagnosis of MAS. In 2016, the European League Against Rheumatism (EULAR)/American College of Rheumatology (ACR)/Pediatric Rheumatology International Trials Organization (PRINTO) classification criteria for MAS were developed [4]. One study by Sung Soo Ahn et al. investigated the application of these new criteria and revealed that around 56% of hospitalized AOSD patients with fever were considered to have MAS, while only 2.86% of AOSD patients were considered to have MAS in an outpatient setting. This suggested that using this classification criteria in an inpatient setting could have skewed the results towards having MAS [5]. This can also explain why the incidence of MAS significantly increased in our NIS 2016–2019 (6.19%) compared to Mehta’s NIS 2009–2013 study (1.7%) [3].

Hospitals with large bed sizes also had increased odds of longer LOS and TOTCHG, which is likely to be due to those institutions having more medically complex patients. It is unclear why our study found that Native Americans had an adjusted decrease in the LOS and TOTCHG compared to White patients. It is possible that these results were skewed by outliers, as only a small percentage of Native Americans were found to have AOSD (0.32%). In fact, the percentage of Native American patients who were admitted with a primary diagnosis of AOSD was 0% from 2016–2018. Furthermore, it is also possible that the Native American patients had milder disease activity, as this race is not known to have increased risk of AOSD.

Currently, the ethnic and racial disparities in AOSD are not well established. One Italian study performed by Franchini et al. in 2010 found that the clinical presentation of AOSD appeared to vary based on the patient’s ethnic background. The results of this study, however, do not accurately represent the population as they were derived from several single-center cohort studies with small sample sizes of less than 90 patients [6].

Other factors, like treatment costs, can also increase the total charge for AOSD patients over the years. One Italian study performed by Ravasio et al. in 2020 found that treating AOSD patients with canakinumab and tocilizumab was less cost-effective than treatment with anakinra [7]. This, however, was an outpatient study and does not sufficiently explain the increased cost found in our study. Moreover, patients who are admitted for AOSD, either as a first presentation or an exacerbation, are typically initially treated with glucocorticoids or non-steroidal anti-inflammatory drugs (NSAIDs) rather than biologics [8]. The exact treatments the patients received are beyond the scope of the NIS database, so further studies need to be performed to determine the impact of treatment on in-patient costs for AOSD patients.

Our findings regarding inpatient mortality for AOSD patients also contrast with to other studies. For example, our NIS 2016–2019 study found that the overall inpatient mortality was 0.929% while Mehta’s NIS 2009–2013 study revealed an overall inpatient mortality of 2.6% [3]. One reason for this discrepancy can be due to advancements made in AOSD treatments after 2012. One meta-analysis, which reviewed all published articles from January 2000 to December 2012 on AOSD treatments, found that, up until 2011, much of the data on AOSD treatment was derived from observational studies, case series, or single case reports [9]. The years 2012 and 2015 were the first in which randomized control trials were performed for the use of Anakinra and Tocilizumab in AOSD, respectively [10,11]. Furthermore, the first ever biologic to receive approval from the United States Food and Drug Administration (FDA) was Canakinumab in 2020 [12]. Advancements in treatment can also explain why our study revealed a substantial decrease in the total number of AOSD admissions (1615 patients over the 4-year period) compared to Mehta’s 2009–2013 study (5820 patients over the 5-year period) [3]. It is also possible that more AOSD cases, either exacerbations or refractory cases, are being treated in an outpatient setting, which can also explain the decrease in AOSD admissions.

In our study, age was the only independent factor for in-patient mortality after adjusting for other variables. Our results revealed that for every 1-year increase in age, there was an 8% decrease in the odds of mortality. Furthermore, all 15 of the AOSD patients that died were between 16 and 35 years old, which is consistent with findings from other studies [13]. Our data also revealed that race did not impact in-patient mortality (aOR = 1) unlike Mehta’s study, which revealed that the odds of in-hospital mortality were 6.39 times higher in Asian patients compared to White patients [3]. As mentioned previously, the current studies on the effects of ethnicities, race, and genetics on AOSD lack consistency and require additional investigation.

## 5. Limitations and Strengths

This study has several limitations. The NIS does not contain unique patient identifiers, so recurrent hospitalizations or hospital transfers can appear as separate observations. Our analysis also relies on ICD-10 codes that increase the risk of coding errors and misclassification of diagnoses. For example, it is possible that patients who had adult transition cases of systemic juvenile idiopathic arthritis were not labeled as having a primary diagnosis of AOSD with the ICD-10 code of M06.1 during their hospital stay.

The NIS dataset also does not contain outpatient data on AOSD, which may have provided a more comprehensive understanding of the disease. The NIS also lacks data on treatment regimens including immunosuppressive agents, imaging studies, and the severity and duration of AOSD prior to hospitalization, which can affect the findings in our study. The NIS also lacks data on how the patients with diagnosed with AOSD, given that it can have several different presentations. This can mean that it is possible that patients can be over- or under-diagnosed with AOSD since there is no exact criteria to diagnose a patient. Other factors, like elevated erythrocyte sedimentation rate (ESR) and C-reactive protein (CRP), can also impact the results of our study; however, these are not commonly given an ICD-10 codes by healthcare professions in an inpatient setting, so the decision was made to not include this as a variable.

Our data may have also overestimated the number of patients with AOSD, as it can often mimic other conditions, like malignancy and sepsis. Although we did exclude patients with other rheumatic conditions from our analysis, we did not exclude those with a primary or secondary diagnosis of sepsis or malignancy, as these can co-exist with an AOSD diagnosis. Furthermore, the NIS does not identify individual patients, so patients who have had recurrent hospitalization stays or were transferred between hospitals can appear as distinct observations.

We also did not include patients with a secondary diagnosis of AOSD, as it is possible that they can be admitted due to another diagnosis that is not related to their condition. This, however, also means that our data did not include patients with a secondary diagnosis of AOSD who may have been admitted due to a complication of AOSD, such as infection, which could change the findings of our study.

This study also has strengths. Our study includes a large sample size, which allows for a more precise estimation of trends and predictors of inpatient outcomes compared to the small cohort studies on AOSD.

## 6. Conclusions

We used our research to highlight the predictors of in-hospital mortality, length of stay, and total charges and provide valuable insights into the resource utilization patterns for AOSD patients. Our results revealed an overall decrease in mortality and in-hospital admissions for a primary diagnosis of AOSD, which is likely to be explained by advances in AOSD treatment, leading to more outpatient management. Our data also revealed that the economic burden of AOSD continues to increase as the in-hospital charge found in our NIS 2016–2019 study (68,415.31 USD) was almost doubled compared to the in-hospital charge found in Mehta’s NIS 2009–2013 study (30,857 USD).

MAS and DIC were both independent factors that impacted the total in-hospital charge. Additionally, the rate of MAS substantially increased from 2009–2013, indicating that new strategies need to be evaluated to prevent these complications. Additionally, our study presents contrasting data regarding race compared to previous AOSD studies and highlights the need for further investigation into its epidemiology and genetics.

## Figures and Tables

**Table 1:** International Classification of Diseases, Tenth Edition, Clinical Modification and Procedure Coding System (ICD-10 CM) Codes used in this study.

Variable	ICD-10 CM Code
Rheumatoid Arthritis	M06.9
Polymyalgia Rheumatica	M35.3
Arthropathic Psoriasis	L40.5
Systemic Lupus Erythematosus	M32.0, M32.10, M32.11, M32.12, M32.13, M32.14, M32.15, M32.19, M32.8, M32.9
Ankylosing Spondylitis	M45.0, M45.1, M45.2, M45.3, M45.4, M45.5, M45.6, M45.7, M45.8, M45.9, M45.A0, M45.A1, M45.A2, M45.A3, M45.A4, M45.A5, M45.A6, M45.A7, M45.A8, M45.AB
Systemic Sclerosis	M34.0, M34.1, M34.2, M34.81, M34.82, M34.83, M34.89, M34.9
Dermato-polymyositis	M33.00, M33.01, M33.02, M33.03, M33.09, M33.10, M33.11, M33.12, M33.13, M33.19, M33.20, M33.21, M33.22, M33.29, M33.90, M33.91, M33.92, M33.93, M33.99
Adult-Onset Still’s Disease	M06.1
Macrophage Activating Syndrome	D76.1
Disseminated Intravascular Coagulation	D65
Thrombotic Thrombocytopenic Purpura	M31.1

**Table 2:** Abbreviations used in this Study.

	Abbreviation
Adult-Onset Still’s Disease	AOSD
National Inpatient Sample	NIS
Total Charge	TOTCHG
Length of Stay	LOS
Macrophage Activating Syndrome	MAS
Disseminated Intravascular Coagulation	DIC
Thrombotic Thrombocytopenic Purpura	TTP

**Table 3:** Baseline characteristics of Adult-Onset Still’s Disease Hospitalizations from 2016–2019.

Patient and Hospital Variables	2016	2017	2018	2019	Total
Observation (n)	360	390	440	425	1615
Mean Age (Years)	43.26	42.53	42.43	43.09	42.54
Sex (%)					
Female	62.50%	60.26%	54.54%	65.88%	60.68%
Male	37.50%	39.74%	45.45%	34.12%	39.32%
Race (%)					
White	53.62%	53.33%	43.68%	50.60%	50%
Black	8.70%	12.00%	20.69%	13.25%	14.01%
Hispanic	18.84%	20.00%	18.39%	21.69%	19.75%
Asian or Pacific Islander	11.59%	9.33%	10.34%	7.23%	9.55%
Native American	0%	0%	0%	1.20%	0.32%
Other	7.25%	5.33%	6.90%	6.02%	6.37%
Hospital Bed Size (%)					
Small	16.67%	6.41%	13.64%	14.12%	12.69%
Medium	20.83%	28.21%	19.32%	15.29%	20.74%
Large	62.50%	65.38%	67.05%	70.59%	66.56%
Hospital location (%)					
Rural	4.17%	5.13%	4.55%	3.53%	4.33%
Urban non-teaching	1.11%	14.10%	6.82%	7.06%	9.60%
Urban teaching	84.72%	80.77%	88.64%	89.41%	86.07%
Hospital Region (%)					
Northeast	31.94%	24.36%	30.68%	25.88%	28.17%
Midwest	20.83%	10.26%	18.18%	21.18%	17.65%
South	27.78%	43.59%	31.82%	27.06%	32.51%
West	19.44%	21.79%	19.32%	25.88%	21.67%
In-Hospital LOS	7.14	7.91	7.33	7.00	7.34
In-Hospital TOTCH	58,575.67 USD	76,051.32 USD	70,365.64 USD	67,723.78 USD	68,415.31 USD

**Table 4:** Univariate and Multivariate Linear Regression of Relationship of Demographic Variables to Hospital Length of Stay.

Clinical Outcomes	Unadjusted OR (95% CI)	*p*-Value for Unadjusted OR	Adjusted OR (95% CI)	*p*-Value for Adjusted OR
Age	0.02 (−0.31 to 0.74)	0.415	−0.01 (−0.07 to 0.05)	0.738
Female	−0.71 (−2.13 to 0.70)	0.325	−0.64 (−2.04 to 7.61)	0.370
MAS	8.64 (1.56 to 15.73)	0.017	8.99 (1.71 to 16.29)	0.016
DIC	8.07 (3.06 to 13.08)	0.002	7.06 (3.87 to 10.24)	0.000
TTP	−2.36 (−6.59 to 1.88)	0.275	−2.92 (−7.43 to 1.69)	0.214
Small Hospital Size	Reference		Reference	
Medium Hospital Size	0.60 (−1.68 to 2.88)	0.607	1.07 (−1.21 to 3.35)	0.303
Large Hospital Size	2.49 (0.68 to 4.29)	0.007	2.24 (0.02 to 4.46)	0.042
White	Reference		Reference	
Black	1.73 (−1.59 to 5.06)	0.307	1.17 (−1.94 to 4.28)	0.461
Hispanic	0.52 (−1.08 to 2.12)	0.527	0.05 (−1.73 to 1.83)	0.953
Asian or Pacific Islander	1.04 (−1.38 to 3.47)	0.398	0.58 (−1.81 to 2.97)	0.636
Native American	−2.79 (−3.78 to −1.80)	0.000	−3.99 (−7.29 to −0.68)	0.018
Other	1.41 (−0.78 to 3.60)	0.207	0.77 (−1.8 to 3.3)	0.557

**Table 5:** Univariate and Multivariate Linear Regression of Relationship of Demographic Variables to Total Hospital Charge.

Clinical Outcomes	Unadjusted OR (95% CI)	*p*-Value for Unadjusted OR	Adjusted OR (95% CI)	*p*-Value for Adjusted OR
Age	−154.51 USD (−888.45 to 579.64)	0.680	−451.73 USD (−1241.143 to 337.680)	0.262
Female	−8059.47 USD (−24,504.54 to 8385.59)	0.337	−11,731.34 USD (−26,403.87 to 2941.182)	0.117
MAS	96,918.06 USD (2295.40 to 191,540.7)	0.045	106,711.80 USD (8702.64 to 204,720)	0.033
DIC	91,840.39 USD (26,800.68 to 156,880.1)	0.006	70,001.37 USD (40,274.57 to 99,728.18)	0.000
TTP	−19,576.54 USD (−59,495.99 to 20,342.91)	0.336	−17,632.92 USD (−67,406.69 to 32,140.84)	0.487
Small Hospital Size	Reference		Reference	
Medium Hospital Size	35,201.61 USD (−884.8402 to 71,288.08)	0.056	13,013 USD (−8626.756 to 34,652.75)	0.239
Large Hospital Size	44,539.42 USD (31,073.82 to 58,005.02)	0.000	29,572.07 USD (6724.683 to 52,419.46)	0.011
White	Reference		Reference	
Black	18,504.43 USD (−26,598.65 to 63,607.50)	0.421	19,247.78 USD (−21,198.44 to 59,694)	0.351
Hispanic	23,948.93 USD (5945.39 to 41,952.47)	0.009	14,824.88 USD (−5786.466 to 35,436.230)	0.159
Asian or Pacific Islander	17,954.29 USD (−18,830.67 to 54,739.25)	0.339	15,215.68 USD (−20,071.48 to 50,502.84)	0.398
Native American	−34,308.14 USD (−43,211.43 to −25,404.85)	0.000	−47,372.49 USD (−85,894.17 to −8850.811)	0.016
Other	23,103.69 USD (−3888.60 to 50,095.98)	0.093	6196.156 USD (−27,446.61 to 39,838.92)	0.718

**Table 6:** Unadjusted and Adjusted Odds Ratio for Independent Predictors of In-Hospital Mortality in Adult-Onset Still’s Disease.

Clinical Outcomes	Unadjusted OR (95% CI)	*p*-Value for Unadjusted OR	Adjusted OR (95% CI)	*p*-Value for Adjusted OR
Age	0.92 (0.87 to 0.98)	0.005	0.85 (0.76 to 0.96)	0.007
Female	0.32 (0.03 to 3.6)	0.355	0.53 (0.014 to 20.21)	0.73
MAS	33.56 (2.90 to 388.07)	0.005	1.00	
DIC	1.00		1.00	
TTP	159.50 (7.17 to 3547.65)	0.001	1.00	
White	Reference		Reference	
Black	1.00		1.00	
Hispanic	1.00		1.00	
Asian or Pacific Islander	1.00		1.00	
Native American	1.00		1.00	

## Data Availability

The datasets used and/or analyzed during the current study are available from the corresponding author on reasonable request.
